# A stable nanoliposomal carboxymethyl cellulose hydrogel of *Pelargonium* essential oil: Formulation, characterization, and enhanced repellent activity against *Anopheles stephensi*

**DOI:** 10.1016/j.parepi.2026.e00490

**Published:** 2026-03-03

**Authors:** Mohsen Kalantari, Kourosh Azizi, Raziyeh Shahheidari, Mahmoud Osanloo, Amir Hossein Roozitalab, Mohsen Fakhraei

**Affiliations:** a Reseach Center for Health Sciences, Institute of Health, Department of Biology and Control of Disease Vectors, School of Health, Shiraz University of Medical Science, Shiraz, Iran; b Student Research Committee, Department of Biology and Control of Disease Vectors, School of Health, Shiraz University of Medical Sciences, Shiraz, Iran; c Department of Medical Nanotechnology, School of Advanced Technologies in Medicine, Fasa University of Medical Sciences, Fasa, Iran

**Keywords:** Pelargonium essential oil, Liposomes, Hydrogels, Mosquito repellents, Anopheles, Plant oils, Nanotechnology, Drug delivery systems

## Abstract

The development of effective botanical repellents is a key strategy in reducing reliance on synthetic compounds such as N,N-Diethyl-meta-toluamide (DEET). This study reports the design and evaluation of a topical nanoliposomal hydrogel incorporating *Pelargonium graveolens* essential oil (PEO) for repellency against *Anopheles stephensi*. Nanoliposomes were prepared using ethanol injection, loaded with 3% PEO, and incorporated into a carboxymethyl cellulose (CMC) hydrogel. The formulation was characterized by dynamic light scattering (DLS), zeta potential, Fourier Transform Infrared (FTIR) spectroscopy, and viscosity analysis. Repellent efficacy was assessed using the arm-in-cage method against unfed female *An. stephensi*, with complete protection time (CPT) recorded for the nanoliposomal gel (LipoGel 3%), compared to free PEO (3%), DEET (3%), and a blank gel. PEO was characterized by Gas Chromatography-Mass Spectrometry (GC–MS), with β-citronellol (30.35%) and geraniol (15.54%) as major constituents. LipoGel 3% displayed a mean particle size of 101 ± 0 nm, a narrow size distribution (Span = 0.95), and a zeta potential of −58.63 ± 0.83 mV. The gel exhibited shear-thinning behavior and remained physically stable for six months. In repellent assays, LipoGel 3% provided a CPT of 216 ± 25 min, significantly longer than free PEO (148 ± 23 min) and the blank gel (33 ± 0 min), though shorter than DEET 3% (311 ± 37 min). Encapsulation of *Pelargonium* essential oil in a nanoliposomal hydrogel significantly prolongs repellent activity against *An. stephensi*. The formulation presents a stable, natural-based topical repellent worthy of further field assessment.

## Highlights

• Nanoliposomal hydrogel delivers *Pelargonium* oil, providing >3.5 h complete protection against *Anopheles stephensi*.
• Geranium oil major components are β-citronellol (30.35%) and geraniol (15.54%), key for repellent activity.
• Formulation shows excellent six-month stability at 4 °C and 26 °C with desirable shear-thinning rheology.
• Encapsulation boosts repellent duration by 46% compared to free essential oil, bridging the gap with DEET.
• Simple ethanol-injection method yields stable, nanoscale vesicles (∼101 nm) with high negative zeta potential.

## 1 Introduction

Malaria persists as a preeminent global health crisis. According to the World Malaria Report 2024, the World Health Organization (WHO) estimates there were 263 million cases and 597,000 deaths globally in 2023. The WHO African Region continues to bear the heaviest burden, accounting for approximately 94% of cases and 95% of deaths worldwide. Children under five remain disproportionately affected, representing 73.7% of all malaria deaths in 2023 (World Health Organization, 2023). This parasitic disease, caused by *Plasmodium* protozoa, is exclusively vectored by female *Anopheles* mosquitoes during a blood meal. The formidable challenge of malaria control is compounded by the biological complexity of its vectors, widespread insecticide resistance, and socio-economic factors limiting the reach of core interventions (Hemingway et al., 2016). Among the numerous anopheline species, *Anopheles stephensi* presents a particularly pressing concern. Historically the primary urban malaria vector across the Indian subcontinent and the Middle East, its invasive spread into the Horn of Africa marks a significant epidemiological shift (Sinka et al., 2010; Balkew et al., 2021). This species' adaptability to both clean and polluted water in urban settings, combined with its efficient transmission capability, threatens to instigate urban malaria outbreaks in regions with historically low transmission and limited immunity, potentially reversing decades of progress (Sinka et al., 2020).

The cornerstone of malaria prevention has long relied on vector-targeted strategies, primarily insecticide-treated nets (ITNs) and indoor residual spraying (IRS). While these tools have been instrumental in reducing transmission, their efficacy is increasingly undermined by the rapid evolution and spread of physiological and behavioral resistance to public health insecticides, most notably pyrethroids (Hemingway et al., 2016). Furthermore, these interventions are inherently limited to protecting individuals indoors during sleeping hours, leaving populations vulnerable to outdoor and early-evening biting by mosquitoes like *An. stephensi* (Killeen et al., 2017). This protection gap underscores the critical, complementary role of personal protective measures, among which topical repellents are paramount. Effective repellents provide a portable, flexible barrier that disrupts the host-seeking behavior of mosquitoes, preventing bites and thereby interrupting the transmission cycle at the individual level, especially in outdoor occupational or social settings (Debboun et al., 2014).

For over seven decades, N,N-Diethyl-meta-toluamide (DEET) has remained the most widely used and efficacious synthetic repellent globally. Its mechanism of action involves interfering with insect olfactory receptors, effectively masking human attractants or directly triggering avoidance behaviors (Ditzen et al., 2008). DEET is recognized to exert its effect through both contact (spatial) and vapor-phase repellency, creating a protective barrier that deters mosquitoes both upon attempted skin contact and from a distance. Despite its proven record, DEET is not without significant drawbacks. Dermal and ocular irritation, allergic contact dermatitis, and, in rare instances of overexposure or ingestion, neurological effects have been documented (Rodriguez et al., 2015). Its potent solvent properties can damage plastics, synthetic fabrics, and watch crystals, limiting its practical use. Perhaps most importantly, growing consumer awareness and preference for “green,” natural, and biocompatible products have fueled a strong demand for alternatives to synthetic chemicals (Nerio et al., 2010). This demand is driven not only by perceived safety but also by environmental concerns regarding the persistence and ecological impact of synthetic compounds.

The search for effective botanical repellents has focused extensively on plant essential oils (EOs), complex mixtures of volatile secondary metabolites such as monoterpenes and sesquiterpenes. These compounds are part of a plant's natural defense arsenal against herbivores and pathogens, and many exhibit broad-spectrum insect-repellent, insecticidal, and oviposition-deterrent properties (Isman, 2020). In contrast to DEET, the repellent action of most essential oils, including that from *Pelargonium* species, is predominantly attributed to vapor-phase activity. The volatile compounds create a repellent odor plume that disrupts host-seeking behavior, with a less pronounced or secondary contact repellent effect compared to synthetic agents like DEET. The essential oil derived from *Pelargonium* species, commonly known as geranium oil, is particularly promising. Its repellent activity is primarily attributed to high concentrations of oxygenated monoterpenes like citronellol, geraniol, and linalool, which have demonstrated efficacy against various mosquito species, including *Anopheles* (Müller et al., 2009). These natural compounds are generally regarded as safe (GRAS), biodegradable, and often possess pleasant aromas, enhancing user acceptability.

However, the translation of essential oils from promising laboratory candidates into reliable, long-lasting repellent products faces two fundamental and interconnected physicochemical challenges: high volatility and poor physicochemical stability. The very property that makes EOs rapidly perceptible to insects—their volatility—also causes them to evaporate quickly from the skin surface. This results in an unacceptably short duration of protection, often less than one to two hours for unformulated oils (Turek and Stintzing, 2013). Furthermore, the unsaturated terpenoid constituents are susceptible to oxidation, isomerization, and polymerization when exposed to light, heat, and oxygen. This degradation not only diminishes repellent efficacy but can also lead to the formation of oxidation products with increased potential for skin sensitization (Ben Miri, 2025). Therefore, a delivery system that can control the release rate, protect the active ingredients from environmental degradation, and prolong their residence time on the skin is essential for developing a viable botanical repellent.

Advances in nanotechnology and drug delivery systems offer powerful solutions to these limitations. Nanoliposomes, spherical vesicles consisting of one or more phospholipid bilayers surrounding an aqueous core, have emerged as ideal carriers for bioactive compounds (Bozzuto and Molinari, 2015). Their amphiphilic nature allows for the efficient encapsulation of both hydrophilic and hydrophobic molecules, making them suitable for the complex mixture of compounds in essential oils. By entrapping the volatile oil within the hydrophobic region of the lipid bilayer, nanoliposomes act as microscopic reservoirs. This encapsulation significantly reduces the immediate evaporation of the oil, provides a physical barrier against oxidative degradation, and enables a sustained, controlled release of the active ingredients, thereby potentially extending the protection time (Echeverría et al., 2019). The nanoscale size (typically 50–200 nm) of these vesicles promotes a high surface-area-to-volume ratio, which can enhance interaction with the skin and improve the uniformity of the formulation.

To create a user-friendly and stable topical product, the nanoliposomal dispersion requires incorporation into a suitable base. Hydrogels, three-dimensional networks of hydrophilic polymers capable of retaining large amounts of water, are excellent candidates for this purpose (Calò and Khutoryanskiy, 2015). A hydrogel matrix, such as one formed from carboxymethyl cellulose (CMC), provides several advantages: it is biocompatible, non-greasy, imparts a pleasant cooling sensation, and is easily washed off. More critically for repellent application, it increases the formulation's viscosity, preventing runoff from sweat and ensuring an even, adherent film on the skin. This matrix can act as a secondary depot, further modulating the release of the nanoliposomes and prolonging the overall residence time of the repellent system on the application site (Kumar et al., 2023).

Previous studies have explored nanoliposomal and hydrogel-based delivery of various essential oils for repellency. For example, nanoliposomal formulations of citronella, eucalyptus, and cinnamon oils have shown improved stability and prolonged efficacy compared to their free oils (Sugumar et al., 2014; Osanloo et al., 2022). However, many studies focus on either the nano-encapsulation or the gel formulation separately, or utilize more complex and less scalable preparation methods. There remains a need for detailed physicochemical characterization and repellent evaluation of a straightforward, easily reproducible system combining *Pelargonium* EO-loaded nanoliposomes within a CMC hydrogel matrix. Furthermore, direct comparative assessment of such a hybrid system against both the free essential oil and the synthetic standard DEET, under standardized conditions against a major malaria vector like *An. stephensi*, is not extensively reported.

This study was designed to address this gap. We hypothesize that encapsulating *Pelargonium* essential oil within nanoliposomes and subsequently incorporating them into a CMC hydrogel will significantly enhance the oil's stability and prolong its repellent efficacy against *Anopheles stephensi* beyond that of the free oil. The specific objectives were to: (1) formulate and physicochemically characterize (particle size, zeta potential, stability, rheology) a CMC hydrogel containing PEO-loaded nanoliposomes prepared via a simple ethanol injection method, and (2) evaluate and compare the Complete Protection Time (CPT) of this novel formulation against laboratory-reared *An. stephensi* females using the WHO-recommended arm-in-cage bioassay, with free PEO and DEET serving as benchmarks. This work aims to contribute a stable, effective, and naturally-derived topical repellent option to the integrated toolbox for personal protection against malaria and other mosquito-borne diseases.

## 2 Materials and methods

### 2.1 Chemicals and reagents

*Pelargonium graveolens* essential oil was obtained via hydro-distillation of aerial parts harvested in Fars province, Iran, and served as the active repellent. GC–MS analysis confirmed its chemical composition, revealing a complex profile characteristic of *Pelargonium* oils. The major constituents were β-citronellol (30.35%) and geraniol (15.54%); other significant components included linalool (9.49%), citronellyl formate (7.43%), and bulnesol (3.54%). In total, 48 compounds were identified, which aligns with the typical phytochemical profile of this essential oil.

The following materials were used for formulation and analysis: carboxymethyl cellulose sodium salt (CMC, medium viscosity, degree of substitution ∼0.7, Sigma-Aldrich, St. Louis, MO, USA) as the gelling agent; soy L-α-phosphatidylcholine (lecithin, >99%, Sigma-Aldrich); cholesterol (≥99%, Sigma-Aldrich); and polysorbates (Tween 20 and Tween 80, Sigma-Aldrich). Absolute ethanol (analytical grade) and all other solvents were purchased from Merck KGaA (Darmstadt, Germany). The synthetic repellent standard, N,N-diethyl-meta-toluamide (DEET, 97%), was also obtained from Sigma-Aldrich. Deionized water was produced using a Milli-Q® Advantage A10 water purification system (Merck Millipore, Burlington, MA, USA) and was used for all aqueous preparations.

### 2.2 Mosquito Colony and rearing conditions

All repellency bioassays were conducted using a susceptible laboratory colony of *Anopheles stephensi* (Liston strain), originally obtained from the insectary of Shiraz University of Medical Sciences (SUMS), Iran. Mosquitoes were reared and maintained under strictly controlled environmental conditions in an insectary, as per standardized protocols (World Health Organization, 2022). The rearing room was maintained at a temperature of 27 ± 2 °C and a relative humidity of 75 ± 10%, with a photoperiod of 12:12 h (light:dark). Larvae were reared in enamel trays containing deionized water and fed ad libitum on a finely ground diet of TetraMin® tropical fish flakes. Pupae were collected and transferred to mesh-covered cages (30 × 30 × 30 cm) for adult emergence.

Adult mosquitoes were provided with a 10% (*w*/*v*) sucrose solution ad libitum via cotton wicks. To maintain the colony and for oogenesis, 5–7 day-old adult female mosquitoes were blood-fed twice weekly using an artificial membrane feeding system (Hemotek Ltd., UK) with defibrinated sheep blood. To obtain highly motivated, host-seeking females for bioassays, sugar wicks were removed 12 h prior to testing, while access to water was maintained (Amer and Mehlhorn, 2006). Only unfed, non-gravid females of this age group (5–7 days' post-emergence) were used in all repellency experiments to ensure consistent host-seeking behavior.

### 2.3 Chemical profiling of *Pelargonium* essential oil

The chemical composition of the essential oil was determined by Gas Chromatography-Mass Spectrometry (GC–MS) to confirm the presence of key repellent compounds. Analysis was performed using an Agilent 7890B GC system coupled with an Agilent 5977 A MSD mass spectrometer (Agilent Technologies, Santa Clara, CA, USA). Separation was achieved on an HP-5MS fused silica capillary column (30 m × 0.25 mm i.d., 0.25 μm film thickness). The oven temperature was programmed from 60 °C (held for 1 min) to 240 °C at a rate of 3 °C/min, with a final hold time of 5 min. Helium was used as the carrier gas at a constant flow rate of 1.0 mL/min. The injector temperature was set at 250 °C, and samples were injected in split mode (split ratio 50:1). Mass spectra were recorded in electron impact (EI) mode at 70 eV, scanning from *m/z* 40 to 550. Identification of components was based on a comparison of their retention indices (RI), calculated relative to a homologous series of n-alkanes (C8–C30), and their mass spectra with those stored in the National Institute of Standards and Technology (NIST 17) library and with published data (Adams, 2001).

### 2.4 Formulation of Nanoliposomes via ethanol injection

Nanoliposomes loaded with *Pelargonium* essential oil (PEO) were prepared using the well-established ethanol injection method, known for its simplicity and reproducibility in generating small, uniform vesicles (Jaafar-Maalej et al., 2010). Initially, a lipid stock solution was prepared by dissolving soy lecithin and cholesterol at a fixed weight ratio of 6:1 (15% *w*/*v* and 2.5% w/v, respectively) in absolute ethanol. This mixture was magnetically stirred at 700 rpm for 24 h at room temperature (25 ± 1 °C) to ensure complete dissolution and molecular interaction.

For the preparation of PEO-loaded nanoliposomes (LipoGel 3%), 1 mL of the clear lipid stock solution was transferred to a glass vial. To this, *Pelargonium* essential oil was added to achieve a final concentration of 3% (*w*/*v*) in the final aqueous dispersion. This concentration was selected based on preliminary repellency screenings and literature reports indicating that concentrations of 3–5% for topical essential oil formulations often provide effective repellency while minimizing the risk of dermal irritation, a critical consideration for natural product-based repellents (Nerio et al., 2010; Amer and Mehlhorn, 2006). Subsequently, a mixed surfactant system of Tween 20 and Tween 80 (1:1 ratio) was incorporated at a total concentration of 1.5% (w/v) to stabilize the forming vesicles and optimize particle size distribution (Sharma et al., 2021). The organic phase was then injected rapidly using a 1 mL glass syringe (Hamilton, USA) into 4 mL of vigorously stirred (1000 rpm) deionized water maintained at 60 °C. The injection was performed dropwise over approximately 1 min. Stirring was continued for an additional 40 min at 60 °C to ensure complete evaporation of ethanol and vesicle maturation. The resulting milky white colloidal suspension was allowed to cool to room temperature. Blank nanoliposomes (LipoGel 0.0%) were prepared following an identical protocol but omitting the addition of PEO.

### 2.5 Preparation of the Carboxymethyl cellulose (CMC) hydrogel matrix

A hydrogel base was prepared using carboxymethyl cellulose (CMC) to incorporate the nanoliposomal dispersion for topical application. A 3.5% (*w*/*v*) CMC hydrogel was formulated using a cold method to prevent lump formation (Clementino et al., 2021). Briefly, the calculated amount of CMC powder was slowly sprinkled onto the surface of ice-cold deionized water under continuous magnetic stirring at 800 rpm. The mixture was stirred for 2 h at room temperature until a clear, viscous, and homogeneous gel formed, free of air bubbles. The gel was then left to hydrate fully overnight at 4 °C to achieve maximum viscosity and clarity.

To obtain the final topical formulation, the freshly prepared nanoliposomal suspension (either blank or PEO-loaded) was gently incorporated into the CMC hydrogel at a 1:1 (*v*/v) ratio. This was done under slow mechanical stirring (200 rpm) for 15 min using an overhead stirrer (Heidolph, Germany) to ensure a homogeneous distribution of the vesicles within the gel matrix without compromising their integrity.

### 2.6 Physicochemical characterization of the formulations

#### 2.6.1 Particle size, polydispersity, and zeta potential

The mean hydrodynamic diameter (*Z*-average), polydispersity index (PDI), and size distribution of the nanoliposomal dispersions were analyzed by Dynamic Light Scattering (DLS) using a Zetasizer Nano ZS instrument (Malvern Panalytical, Malvern, UK) at a fixed scattering angle of 173° and a temperature of 25 °C. Samples were appropriately diluted with filtered (0.22 μm) deionized water prior to analysis to avoid multiple scattering effects. The Span value, indicating the width of the particle size distribution, was calculated as (d90 - d10) / d50, where d10, d50, and d90 are the particle diameters at 10%, 50%, and 90% cumulative volume, respectively (Danaei et al., 2018). The zeta potential, a key indicator of colloidal stability, was measured using the same instrument via laser Doppler micro-electrophoresis. All measurements were performed in triplicate.

#### 2.6.2 Attenuated Total reflectance-Fourier transform infrared (ATR-FTIR) spectroscopy

Chemical characterization and verification of successful encapsulation were performed using ATR-FTIR spectroscopy (Thermo Scientific Nicolet iS10, USA). Spectra were recorded in the range of 400–4000 cm^−1^ with a resolution of 4 cm^−1^ and 32 scans per sample. Samples analyzed included pure PEO, plain CMC hydrogel, blank nanoliposomal hydrogel (LipoGel 0.0%), and the PEO-loaded nanoliposomal hydrogel (LipoGel 3%).

#### 2.6.3 Rheological analysis

The viscosity and flow behavior (rheology) of the prepared hydrogels were assessed using a rotational rheometer (MCR 302, Anton Paar, Austria) equipped with a parallel-plate geometry (plate diameter 25 mm, gap 1 mm) at 25 °C. Flow curves were obtained by measuring the apparent viscosity (η) while logarithmically increasing the shear rate from 0.1 to 100 s^−1^. The data were analyzed to determine if the gels exhibited shear-thinning (pseudoplastic) behavior, which is desirable for easy application and good skin adherence (Bruschi, 2015).

#### 2.6.4 Physical stability study

The short-term physical stability of the final hydrogels (LipoGel 0.0% and LipoGel 3%) was evaluated by visual inspection for any signs of phase separation, creaming, sedimentation, or color change. Samples were stored in sealed glass vials for six months at two different temperatures: refrigerated conditions (4 ± 1 °C) and room temperature (26 ± 1 °C). Observations were recorded weekly for the first month and monthly thereafter.

Following the six-month storage period, samples stored at both temperatures were re-evaluated for key parameters to confirm the retention of functional stability. The mean particle size, zeta potential, and rheological profile (as described in sections 2.6.1 and 2.6.3) were measured and compared to the values obtained for the freshly prepared formulations. Most critically, the repellent efficacy (Complete Protection Time, CPT) of the stored LipoGel 3% was re-assessed using the arm-in-cage bioassay (as per section 2.7) against *An. stephensi*.

### 2.7 Repellent bioassay: Arm-in-cage method

**Fig. 1:**
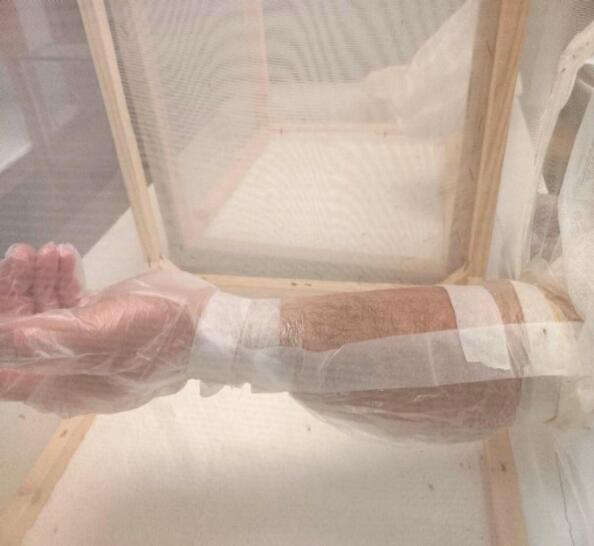
Arm-in-cage assay under controlled insectary conditions.

The repellent efficacy of the formulations was evaluated against unfed female *An. stephensi* using the human-bait arm-in-cage method, following WHO guidelines with minor modifications (World Health Organization, 2009). The study protocol received full ethical approval from the Institutional Review Board of SUMS (Approval ID: IR.SUMS.AEC.1403.038). Written informed consent was obtained from all participating adult volunteers (*n* = 3). Volunteers were instructed to refrain from using perfumes, scented soaps, lotions, or smoking for at least 12 h prior to testing (Fig. 1).

On the day of the assay, the volunteer's forearm was washed with odorless soap, rinsed thoroughly, and wiped with a 70% ethanol solution, then allowed to dry completely. A rectangular area of 25 cm^2^ (5 cm × 5 cm) on the ventral side of the forearm was delineated. A dose of 1 μL of test formulation per cm^2^ of skin (i.e., 25 μL total) was applied evenly within this area using a micropipette. The following test samples were evaluated: (1) LipoGel 3%, (2) LipoGel 0.0% (negative control), (3) free *Pelargonium* essential oil (3% *v*/v in ethanol), and (4) DEET (3% v/v in ethanol, positive control). The hand was protected with an odorless latex glove.

Five minutes after application, the treated forearm was inserted into a cubic cage (40 × 40 × 40 cm) containing 200 hungry, unfed female *An. stephensi*. The exposure lasted for 3 min, during which the number of landings and confirmed bites (proboscis insertion) were recorded by a trained observer. The arm was withdrawn after 3 min. This procedure was repeated every 30 min until the first confirmed bite occurred on the treated area. The CPT was defined as the time interval (in minutes) between application and the first confirmed bite. Each formulation was tested in triplicate on three different days using different batches of mosquitoes and a rotating panel of volunteers to minimize bias.

### 2.8 Statistical analysis

All assays were conducted with a minimum of three independent replicates (*n* ≥ 3). Data are presented as mean ± standard deviation (SD). Statistical analysis was performed using SPSS software (Version 27.0, IBM Corp., Armonk, NY, USA). The significance of differences in the CPT among the different treatment groups was determined using a one-way analysis of variance (ANOVA). Following a significant ANOVA result, multiple comparisons between group means were conducted using Tukey's Honestly Significant Difference (HSD) post-hoc test. A *p*-value of less than 0.05 was considered statistically significant for all tests (McDonald, 2014).

## 3 Results

### 3.1 Chemical profiling of *Pelargonium graveolens* essential oil

**Fig. 2:**
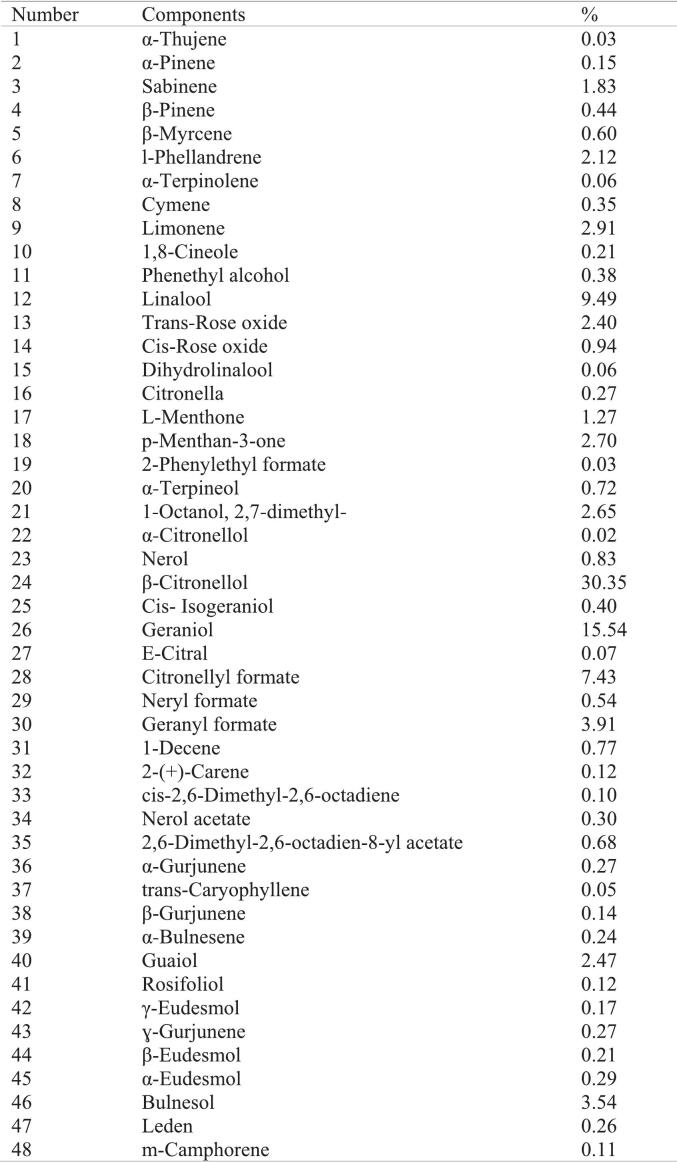
List of components identified in Pelargonium using GC–MS analysis.

Gas Chromatography-Mass Spectrometry (GC–MS) analysis of the hydro-distilled *Pelargonium graveolens* essential oil confirmed a complex phytochemical profile consistent with its documented repellent properties. A total of 48 volatile constituents were identified, comprising approximately 99.2% of the total oil composition. The profile was dominated by oxygenated monoterpenes, which are widely recognized for their insect-repellent activities. The major constituents were β-citronellol (30.35%) and geraniol (15.54%), collectively accounting for nearly 46% of the total oil. Other significant components included linalool (9.49%), citronellyl formate (7.43%), and bulnesol (3.54%). Minor constituents comprised additional monoterpenes, sesquiterpenes, and aromatic compounds (Fig. 2). This composition aligns with previous chemotypic reports for geranium oil, which highlight citronellol and geraniol as key bioactive compounds responsible for interfering with mosquito olfactory reception (Luker et al., 2023; Al-Mijalli et al., 2022).

### 3.2 Physicochemical characterization of Nanoliposomal dispersions

The nanoliposomal dispersions were successfully prepared using the ethanol injection method. Dynamic light scattering (DLS) analysis revealed that the PEO-loaded nanoliposomes (LipoGel 3%) exhibited a mean hydrodynamic diameter of 101 ± 4 nm with a narrow size distribution, evidenced by a Span value of 0.95. In contrast, blank nanoliposomes (LipoGel 0.0%) showed a larger mean diameter of 172 ± 14 nm and a broader distribution (Span >1), indicating that the incorporation of the essential oil influenced vesicle assembly and size homogeneity. The reduction in size for the loaded formulation is advantageous for skin permeation and stability (Akbarzadeh et al., 2013). Zeta potential measurements, critical for predicting colloidal stability, demonstrated strong negative surface charges of −58.63 ± 0.83 mV for LipoGel 3% and − 51.88 ± 0.80 mV for the blank formulation. These high negative values, attributable to the anionic phospholipid headgroups, suggest excellent electrostatic repulsion between vesicles, thereby minimizing aggregation and ensuring long-term physical stability (Ghasemiyeh and Mohammadi-Samani, 2020).

### 3.3 Spectroscopic confirmation and compatibility

**Fig. 3:**
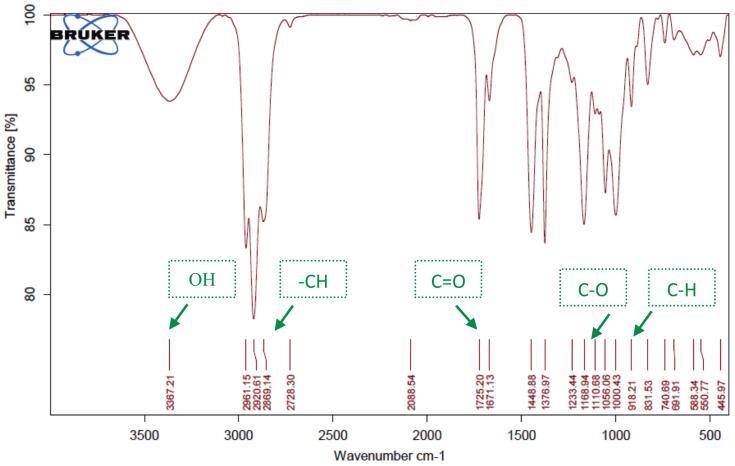
FTIR spectra of *Pelargonium* EO displayed, the broad peak at 3368 cm-1related to OH due to phenolic compound in EO, the stretching vibration at 2961, 2920 and 2869 cm-1 showed –CH, the peak at1725 and 1671 cm-1 related to carbonyl C=O groups in EO. Absorption band at 1448 cm-1 is assigned to the aromatic ring CH2, the peak at 1376 cm-1 is related to present of CH3, the strong peak at 1168, 1110 and 1000 cm-1 are allocated to the stretching vibrations of C—O and the C-OH deformation vibration. The peak at 918 cm-1 is assigned to C—H bending absorption, and the strong peak at 740 cm-1 is assigned to benzene rings C—H vibration absorption.

**Fig. 4:**
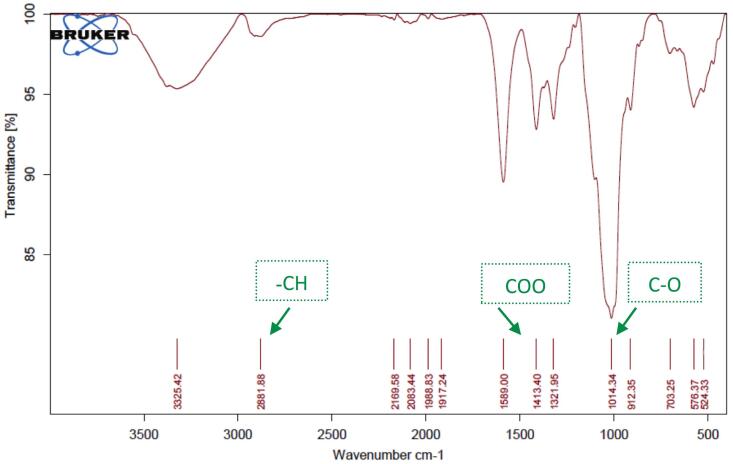
ATR-FTIR spectrum of CMC displayed the broad bands at 3325 cm-1 can be attributed to the stretching of the hydroxyl group due to H-bonding, the absorption at 2881 cm^−1^ showed –CH, the strong absorption at 1589 cm^−1^ related to COO group (asymmetric. Stretching) and the spectra at 1413 cm^−1^ related to COO (symmetric. Stretching) the band at around 1014 allocated to C—O.

**Fig. 5:**
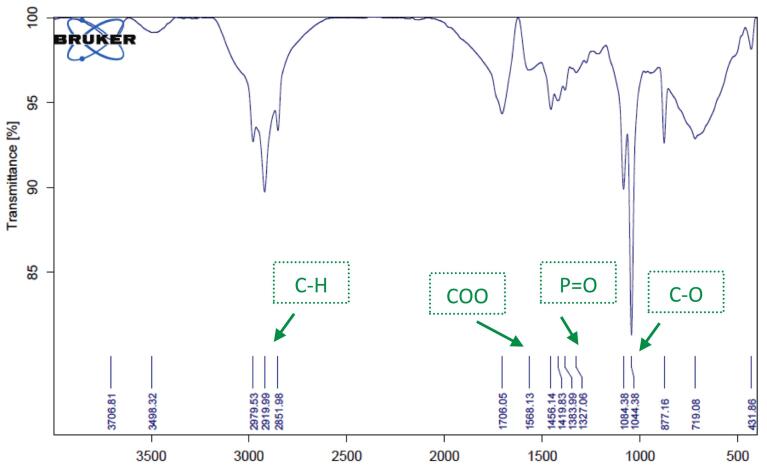
ATR-FTIR of blank nanogel containing free liposome showed the hydroxyl group in 3498 cm^−1^. The absorption at 2979, 2991 and 2851 cm^−1^ assigned to C—H, due to sp^3^ hybrid compounds in cholesterol, Lecithin, CMC and tween, the stretching vibration at 1706 cm^−1^ related to carbonyl group in liposome. The spectra at 1568 cm^−1^ related to asymmetric. Stretching of COO group due to CMC. The spectra at around 1327 cm^−1^ related to the phosphate group in liposome. The absorption at 1084 and 1044 cm^−1^ related to present of C—O.

**Fig. 6:**
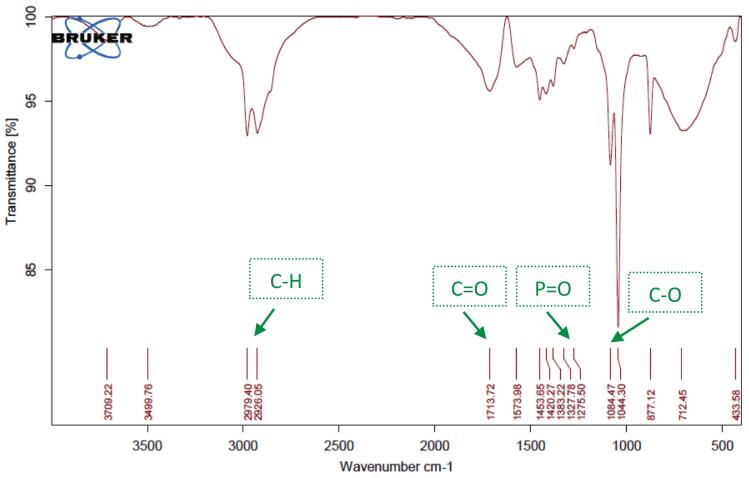
ATR-FTIR of hydrogel containing *Pelargonium* EO in liposome displayed the broad and characteristic band at 3499 cm^−1^ confirmed the present of hydrogen bonding between liposome containing *Pelargonium* EO and hydrogel of CMC. The stretching vibration at 2978 and 2926 and 2855 cm^− 1^connected to C—H. The strong vibration at 1713 cm^− 1^ related to C=O in the ester bonds of the phospholipid, the band at 1275 cm^−1^ related to the phosphate group in phospholipid in liposome, this two characteristic band confirmed the liposome structure, the vibration at 1420 cm^−1^ confirmed the present of carboxylic acid groups (-COOH) in CMC. The sharp vibration at 1084 and 1044 cm^−1^ linked to C—O.

ATR-FTIR spectroscopy (Nicolet iS10, Thermo Fisher Scientific) was employed to verify the successful incorporation of *Pelargonium* essential oil into the nanoliposomal hydrogel matrix (Topală et al., 2015). The spectrum of the pure essential oil displayed characteristic absorption bands at approximately 3368 cm^−1^ (O—H stretching of alcohols), 1725 cm^−1^ (C=O stretching of esters), and prominent peaks in the fingerprint region (1500–900 cm^−1^) corresponding to terpene skeletons. The spectrum of the final LipoGel 3% formulation retained the key carbonyl stretch (shifted to 1713 cm^−1^), confirming the presence of the oil, while also showing distinctive bands from the nanoliposome structure (e.g., P=O stretch at ∼1275 cm^−1^) and the CMC hydrogel matrix (broad O—H stretch and C–O–C vibrations). The absence of new peaks indicated no chemical interaction or degradation, suggesting physical encapsulation and compatibility among all components (Fig. 3, Fig. 4, Fig. 5, Fig. 6).

### 3.4 Rheological behavior and physical stability

The rheological profiles of both the blank and PEO-loaded hydrogels exhibited pseudoplastic (shear-thinning) behavior, a desirable attribute for topical applications. Apparent viscosity decreased progressively as the shear rate increased from 0.1 to 100 s^−1^. This non-Newtonian flow facilitates easy spreading upon application while ensuring the formulation remains adherent on the skin under static conditions (Bruschi, 2015). Physical stability studies conducted over six months under refrigerated (4 °C) and ambient (26 °C) storage conditions showed no visible signs of phase separation, syneresis, color change, or precipitation in either formulation. Consistent with the visual observations, quantitative analyses post-storage confirmed the robustness of the nanoliposomal hydrogel system. For the LipoGel 3% stored at 4 °C and 26 °C, the mean particle size remained unchanged at 102 ± 3 nm and 105 ± 5 nm, respectively, and the zeta potential retained high negative values (−57.2 ± 1.1 mV and − 56.8 ± 1.3 mV, respectively), indicating no significant aggregation or loss of colloidal stability. The shear-thinning rheological behavior was preserved. Importantly, the repellent efficacy of the stored LipoGel 3% was maintained, with CPT values of 210 ± 28 min (4 °C) and 205 ± 30 min (26 °C). These values were not statistically different (*p* > 0.05) from the CPT of the fresh formulation (216 ± 25 min). The consistent macroscopic appearance, physicochemical parameters, and retained biological activity underline the robustness of the nanoliposomal hydrogel system for potential commercial shelf-life (Zhao et al., 2023).

The visual and tactile characteristics of the key formulations were carefully documented. The blank CMC hydrogel was transparent, colorless, and had a smooth, glossy appearance. In contrast, the PEO-loaded nanoliposomal hydrogel (LipoGel 3%) was visually distinct, presenting as an opaque, homogenous, off-white cream with a smooth, non-greasy texture. This marked change in appearance from a transparent gel to an opaque cream provides clear visual evidence of the successful and uniform incorporation of the nanoliposomal dispersion within the CMC hydrogel matrix.

### 3.5 Repellent efficacy against *Anopheles stephensi*

**Fig. 7:**
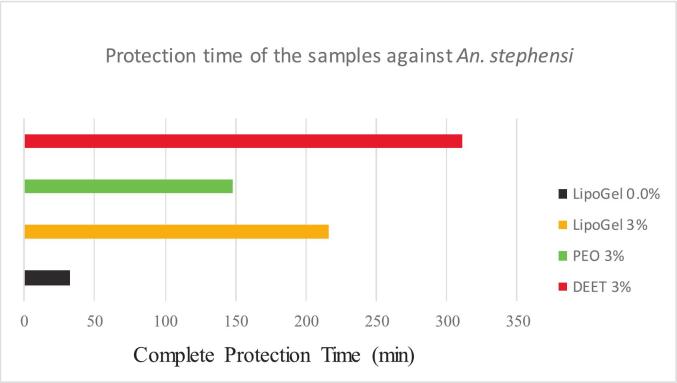
Chart of Complete Protection Time of the samples against *An. stephensi.*

The repellent performance of the formulations was quantitatively assessed via the arm-in-cage bioassay, with CPT serving as the primary endpoint (Fig. 7).

The blank nanoliposomal hydrogel (LipoGel 0.0%) provided negligible protection, with a CPT of only 33 ± 5 min, confirming that the vehicle components lacked inherent repellency. The free *Pelargonium* essential oil (3% in ethanol) offered a CPT of 148 ± 23 min, demonstrating its intrinsic repellent activity but also highlighting its volatility-limited duration. Remarkably, the PEO-loaded nanoliposomal hydrogel (LipoGel 3%) significantly extended the protection time to 216 ± 25 min (*p* < 0.05 compared to free PEO). This represents a 46% increase in efficacy, directly attributable to the encapsulation system's ability to modulate the evaporation rate of the volatile terpenes (Najafzadeh Nansa et al., 2024). As expected, the 3% DEET positive control yielded the longest CPT of 311 ± 37 min, consistent with its established potency. However, the performance of LipoGel 3% positions it as a highly effective natural alternative, bridging a significant gap between conventional botanical repellents and synthetic standards.

## 4 Discussion

This study successfully demonstrates that a nanoliposomal hydrogel delivery system significantly enhances the repellent efficacy and duration of *Pelargonium graveolens* essential oil (PEO) against *Anopheles stephensi*, a major malaria vector. The central finding—that the LipoGel 3% formulation provided 216 min of complete protection, a 46% increase over the free essential oil—validates our hypothesis and underscores the critical role of advanced formulation science in overcoming the inherent limitations of botanical repellents. This enhanced performance is not merely an incremental improvement but represents a meaningful step toward a viable, long-lasting natural alternative to synthetic standards like DEET.

The pronounced repellent activity of the free PEO (148 min) can be directly attributed to its rich composition of oxygenated monoterpenes, primarily β-citronellol and geraniol. These compounds are well-established to interfere with the mosquito's olfactory system by either masking host-derived kairomones (e.g., lactic acid, ammonia) or directly activating specific odorant receptor neurons that elicit avoidance behavior (Luker et al., 2023; Afify et al., 2019). It is important to note that the primary mode of action for such volatile plant terpenes is vapor-phase repellency. However, the volatile nature of these terpenes leads to rapid evaporation from the skin, resulting in the characteristic short-lived protection observed with unformulated essential oils. Our encapsulation strategy directly addresses this Achilles' heel of botanical repellents. The nanoliposomal system acts as a microscopic reservoir, entrapping the volatile oil within its phospholipid bilayers. This structure provides a physical barrier that drastically reduces the immediate evaporation rate, facilitating a sustained, controlled release of the active volatiles at the skin-air interface (Echeverría et al., 2019; Najafzadeh Nansa et al., 2024). The resulting prolonged maintenance of a repellent-effective concentration is the fundamental mechanism behind the extended CPT observed for LipoGel 3%.

The physicochemical data provide robust support for this controlled-release mechanism. The PEO-loaded nanoliposomes exhibited a mean diameter of approximately 101 nm with a narrow size distribution (Span = 0.95). This optimal nanoscale size is crucial, as it provides a high surface-area-to-volume ratio, which favors a sustained release profile and promotes uniform dispersion and adhesion on the skin surface (Danaei et al., 2018). Furthermore, the high negative zeta potential (≈ −59 mV) indicates excellent colloidal stability due to strong electrostatic repulsion between vesicles. This stability is essential for preventing aggregation during storage, ensuring the integrity of the reservoir system, and guaranteeing consistent performance upon application (Ghasemiyeh and Mohammadi-Samani, 2020). The incorporation of these stable nanoliposomes into the CMC hydrogel created a synergistic dual-barrier system. The hydrogel matrix serves a dual purpose: it acts as a secondary macroscopic reservoir that further modulates release kinetics, and its shear-thinning rheology ensures easy application while forming a uniform, adherent film on the skin that resists runoff from sweat or water exposure (Bruschi, 2015; Zhao et al., 2023).

Notwithstanding these promising outcomes, a critical analysis of our design approach reveals limitations that warrant attention in future development. First, the formulation process, while straightforward, involved multiple steps (lipid dissolution, injection, hydrogel incorporation) that could complicate scale-up and increase batch-to-batch variability in an industrial setting. Future designs should consider continuous-flow manufacturing or one-pot synthesis methods to enhance reproducibility and production efficiency. Second, our study focused on a single, fixed concentration of PEO (3%). A more systematic concentration-response study would be necessary to identify the optimal loading that maximizes duration without compromising dermal safety or formulation stability. Furthermore, the use of a mixed surfactant system (Tween 20/80), while effective for stabilization, might not be ideal for all user groups; exploring alternative, skin-friendlier stabilizers or optimizing the phospholipid-to-cholesterol ratio could improve the biocompatibility profile. Finally, the 1:1 (*v*/v) ratio of nanoliposomal dispersion to CMC hydrogel was empirically chosen. A formal design-of-experiments (DoE) approach to optimize this ratio, as well as the total polymer concentration, could yield a formulation with even better textural and release properties.

When contextualized within the existing literature, our findings align with and extend the growing body of evidence supporting nano-encapsulation for repellent delivery. Previous studies have reported similar efficacy enhancements using various nanocarriers. For instance, Sugumar et al. (2014) showed that nanoemulsions of eucalyptus and citronella oils provided longer protection than their free counterparts (Sugumar et al., 2014). More recently, Osanloo et al. (2022) reported a CPT of over 300 min for a cinnamon oil nanoliposomal gel against *An. stephensi*, a result comparable to our DEET control (Osanloo et al., 2022). Furthermore, other studies reinforce the broad applicability of nanoformulations. For example, a nanostructured lipid carrier system loaded with lemongrass oil demonstrated enhanced repellent activity and prolonged release against *Aedes aegypti* (Kechagia et al., 2024). Similarly, solid lipid nanoparticles containing *Zanthoxylum limonella* oil showed improved photostability and repellent efficacy against *Anopheles* min*imus* (Sakulku et al., 2009). Our work complements these studies by providing a detailed characterization of a simple, scalable, and excipient-lean system specifically for geranium oil---a popular yet volatility-plagued repellent. When comparing physicochemical parameters, our nanoliposomes exhibited a favorable particle size (∼101 nm) and exceptionally high negative zeta potential (∼ −59 mV). These values compare well with, or exceed, those reported for other essential oil-loaded liposomal systems designed for topical application. For instance, nanoliposomes containing *Cinnamomum zeylanicum* oil reported sizes of ∼140 nm and zeta potentials of ∼ −40 mV, while formulations with *Zataria multiflora* oil showed sizes around 120 nm and zeta potentials near −30 mV. The superior colloidal stability suggested by our higher zeta potential likely contributes to the excellent physical stability observed over six months.

The novelty of our approach lies in the combination of a straightforward ethanol injection method for liposome preparation with a biocompatible CMC hydrogel, avoiding complex surfactants or solvents that could raise safety or regulatory concerns. All primary excipients—soy lecithin, cholesterol, and CMC—are Generally Recognized As Safe (GRAS), enhancing the translational potential of the formulation for consumer products (Clementino et al., 2021; Sharma et al., 2021).

It is important to critically analyze the performance relative to DEET. While the 3% DEET solution provided the longest CPT (311 min), the LipoGel 3% formulation succeeded in bridging a significant portion of this efficacy gap. This is a notable achievement, as many natural repellent formulations struggle to provide more than 2 h of protection. The remaining performance difference underscores DEET's unparalleled efficiency in interacting with insect olfactory receptors and its dual capacity for both potent vapor-phase and contact repellency (Ditzen et al., 2008). In contrast, the activity of the PEO formulation is likely sustained primarily through the controlled release of vapor-phase actives. However, from a public health and consumer choice perspective, a natural formulation offering close to 4 h of complete protection addresses a substantial segment of outdoor exposure risk, particularly for individuals who avoid DEET due to concerns about skin irritation, damage to synthetics, or personal preference for natural products (Rodriguez et al., 2015; Isman, 2020). Furthermore, the encapsulation strategy could potentially allow for a higher, yet safe, loading of PEO to further extend protection time without causing dermal irritation, a pathway worth exploring in future formulations.

The implications of this research extend beyond the laboratory. The development of a stable, effective, and user-friendly natural repellent has tangible applications in integrated vector management (IVM) strategies (Kumar et al., 2023). In regions where *An. stephensi* is expanding, including urban areas in the Horn of Africa, and where insecticide resistance compromises traditional tools like ITNs and IRS, such topical repellents become a critical complementary intervention (Balkew et al., 2021; Sinka et al., 2020). They offer personal protection during early evening and outdoor activities—key exposure periods not covered by indoor interventions. The six-month physical stability observed under different storage conditions is a promising indicator for shelf-life, a practical necessity for field deployment and commercial viability in resource-limited settings.

Nevertheless, we must acknowledge the limitations inherent in this proof-of-concept study. The bioassays were conducted under controlled laboratory conditions with a single, susceptible mosquito strain. Field evaluations are imperative to assess efficacy against wild, heterogeneous mosquito populations under real-world environmental variables such as wind, high temperatures, and intense humidity, all of which can affect the release rate and longevity of the repellent (Njiru et al., 2006). Furthermore, while the blank formulation showed no irritancy in our limited tests, comprehensive dermatological safety assessments, including repeat-insult patch tests on human volunteers, are required before any human use can be recommended. The specificity to *An. stephensi* also warrants expansion; evaluating the broad-spectrum efficacy against other medically important vectors like *Aedes aegypti* (dengue, Zika) and *Culex quinquefasciatus* (West Nile virus, lymphatic filariasis) is a logical next step (Abrantes et al., 2021).

Future research directions are multi-faceted. First, investigating the precise release kinetics of key terpenes (citronellol, geraniol) from the hydrogel matrix using in vitro Franz diffusion cell studies would provide mechanistic clarity and allow for formulation fine-tuning. Second, scaling up the ethanol injection process for pilot-scale manufacturing is essential for translation. Third, exploring the incorporation of other synergistic essential oils or safe fixatives (like vanillin) within this delivery platform could potentially enhance and further prolong the repellent effect (Nerio et al., 2010). Finally, engaging in community-based field trials in endemic areas will be the ultimate test of this technology's public health value and user acceptability.

Notwithstanding these promising outcomes, a critical analysis of our design approach reveals limitations that warrant attention in future development. First, the formulation process, while straightforward, involved multiple steps (lipid dissolution, injection, hydrogel incorporation) that could complicate scale-up and increase batch-to-batch variability in an industrial setting. Future designs should consider continuous-flow manufacturing or one-pot synthesis methods to enhance reproducibility and production efficiency. Second, our study focused on a single, fixed concentration of PEO (3%). A more systematic concentration-response study would be necessary to identify the optimal loading that maximizes duration without compromising dermal safety or formulation stability. Furthermore, the use of a mixed surfactant system (Tween 20/80), while effective for stabilization, might not be ideal for all user groups; exploring alternative, skin-friendlier stabilizers or optimizing the phospholipid-to-cholesterol ratio could improve the biocompatibility profile. Finally, the 1:1 (*v*/v) ratio of nanoliposomal dispersion to CMC hydrogel was empirically chosen. A formal design-of-experiments (DoE) approach to optimize this ratio, as well as the total polymer concentration, could yield a formulation with even better textural and release properties.

In conclusion, this study provides compelling evidence that the nano-encapsulation of *Pelargonium graveolens* essential oil within a liposomal hydrogel system effectively mitigates its volatility, leading to a significant and practically meaningful extension of repellent efficacy. By narrowing the performance gap with DEET through a biocompatible and stable formulation, this work contributes a promising candidate to the arsenal of personal protective measures against malaria and other mosquito-borne diseases. It exemplifies how rational formulation design can unlock the potential of natural bioactive compounds, moving them from promising laboratory curiosities toward viable, safe, and effective public health tools.

## 5 Conclusion

In summary, this study successfully designed, fabricated, and evaluated an innovative liposome-based gel system for the delivery of *Pelargonium graveolens* essential oil. The developed formulation effectively addressed the principal limitations of volatility and rapid degradation associated with the unprocessed botanical extract. By encapsulating the essential oil within nanoscale phospholipid vesicles with a mean diameter of 101 ± 4 nm and a high negative zeta potential (−58.6 mV) and incorporating them into a carboxymethyl cellulose hydrogel, a significant and sustained enhancement in repellent activity against *Anopheles stephensi* was achieved. The optimized formulation provided 216 ± 25 min (over 3.5 h) of complete protection, which represents a 46% increase (*p* < 0.05) compared to the free essential oil (148 ± 23 min).

This improved efficacy is directly attributable to the dual-function delivery platform. The nanoliposomes serve as a protective reservoir, substantially reducing the immediate evaporation of the oil's active terpenic constituents and facilitating their controlled release at the skin surface. Concurrently, the hydrogel matrix ensures optimal topical application, enhancing skin adherence and providing a secondary depot effect. The formulation demonstrated excellent physicochemical stability over six months at both 4 °C and 26 °C, with no significant change in particle size, zeta potential, rheology, or repellent efficacy (CPT of ∼210 min' post-storage), alongside desirable rheological properties for user comfort, underscoring its practical viability.

Consequently, this work substantiates the potential of integrated nano-formulation strategies to transform promising natural bioactive compounds into reliable, long-lasting personal protective tools. The presented nanoliposomal hydrogel emerges as a compelling, biocompatible alternative to synthetic repellents, contributing a valuable candidate to the portfolio of interventions for mosquito-borne disease prevention. Future investigations should focus on confirmatory field efficacy trials against wild mosquito populations, comprehensive human safety assessments, and the exploration of scalable production processes to facilitate its translation from the laboratory to public health utility.

## Human ethic notes

All subjects gave informed consent for inclusion before participating in the study. “Informed consent” was obtained from all subjects and/or their legal guardians.

## Animal ethic notes

All animal experimental protocols were approved by the Science and Ethics Committee of Shiraz University of Medical Sciences (SUMS).

## CRediT authorship contribution statement

**Mohsen Kalantari:** Writing – review & editing, Writing – original draft, Supervision, Project administration, Conceptualization. **Kourosh Azizi:** Writing – review & editing, Validation, Methodology. **Raziyeh Shahheidari:** Writing – review & editing, Software, Investigation. **Mahmoud Osanloo:** Writing – review & editing, Validation, Methodology. **Amir Hossein Roozitalab:** Investigation, Methodology, Writing – review & editing. **Mohsen Fakhraei:** Writing – review & editing, Visualization, Investigation.

## Ethical approval

This study was conducted in accordance with international, national, and institutional ethical guidelines. We declare that all experiments were performed in accordance with the ARRIVE guidelines 2.0 and that all experimental protocols were approved by Ethical approval obtained from the Science and Ethics Committee of SUMS (Approved ID: IR.SUMS.AEC.1403.038).

## Author contributions

M.K. designed, supervised and wrote the paper. M.O., K.A., R.S. and M.F. performed experiments, analyzed data, and co-wrote the paper.

## Funding

This research was funded by Iran National Science Foundation (INSF, Project no. 40404091) and Shiraz University of Medical Sciences (Grant no. 30319).

## Declaration of competing interest

The authors declare that they have no known competing financial interests or personal relationships that could have appeared to influence the work reported in this paper.
